# Long-term outcomes of single-incision plus one-port laparoscopic surgery versus conventional laparoscopic surgery for rectosigmoid cancer: a randomized controlled trial

**DOI:** 10.1186/s12885-023-11500-2

**Published:** 2023-12-07

**Authors:** Xuehua Zhang, Haitao Yuan, Zilin Tan, Gaohua Li, Zhenzhao Xu, Jinfan Zhou, Jie Fu, Mingyi Wu, Jiafei Xi, Yanan Wang

**Affiliations:** 1 Department of General Surgery, Guangdong Provincial Key Laboratory of Precision Medicine for Gastrointestinal Tumor, The First School of Clinical Medicine, Nanfang Hospital, Southern Medical University, Guangzhou, 510515 Guangdong China; 2 Stem Cell and Regenerative Medicine Lab, Beijing Institute of Radiation Medicine, Beijing, 100850 China

**Keywords:** Long-term outcomes, Single-incision plus one-port, Laparoscopic Surgery, Rectosigmoid cancer, Randomized controlled trial

## Abstract

**Background:**

Though our previous study has demonstrated that the single-incision plus one-port laparoscopic surgery (SILS + 1) is safe and feasible for sigmoid colon and upper rectal cancer and has better short-term outcomes compared with conventional laparoscopic surgery (CLS), the long-term outcomes of SILS + 1 remains uncertain and are needed to evaluated by an RCT.

**Methods:**

Patients with clinical stage T1-4aN0-2M0 rectosigmoid cancer were enrolled. The participants were randomly assigned to either SILS + 1 (n = 99) or CLS (n = 99). The 3-year DFS, 5-year OS, and recurrence patterns were analyzed.

**Results:**

Between April 2014 and July 2016, 198 patients were randomly assigned to either the SILS + 1 group (n = 99) or CLS group (n = 99). The median follow-up in the SILS + 1 group was 64.0 months and in CLS group was 65.0 months. The 3-year DFS was 87.8% (95% CI, 81.6–94.8%) in SILS + 1 group and 86.9% (95% CI, 81.3–94.5%) in CLS group (hazard ratio: 1.09 (95% CI, 0.48–2.47; P = 0.84)). The 5-year OS was 86.7% (95% CI,79.6–93.8%) in the SILS + 1 group and 80.5% (95% CI,72.5–88.5%) in the CLS group (hazard ratio: 1.53 (95% CI, 0.74–3.18; P = 0.25)). There were no significant differences in the recurrence patterns between the two groups.

**Conclusions:**

We found no significant difference in 3-year DFS and 5-year OS of patients with sigmoid colon and upper rectal cancer treated with SILS + 1 vs. CLS. SILS + 1 is noninferior to CLS when performed by expert surgeons.

**Trial registration:**

ClinicalTrials.gov: NCT02117557 (registered on 21/04/2014).

## Introduction

Colorectal cancer is the third most common cancer in China with an estimated 550,000 newly diagnosed cases each year [1]. Conventional laparoscopic surgery (CLS) is a minimally invasive technique that has been accepted as an alternative to traditional open surgery for colorectal cancer due to its comparable short-term benefits and long-term oncological safety [2–9]. However, CLS would normally require 4 or 5 abdominal incisions for trocars and one mini-laparotomy incision for specimen extraction and each incision could be associated with pain and wound complication. Nowadays, to further reduce the surgical trauma, several randomized clinical trials (RCTs) have explored the safety and feasibility of single-incision laparoscopic colectomy (SILC) [10–12]. Though these studies had reported that SILC could achieve more minimally invasive effect compared to CLS, SILC is still technically limited owing to limited instrument movement, loss of triangulation, and poorer in-line viewing. To overcome the obstacles associated with SILC in treatment of rectosigmoid cancer, the single-incision plus one-port laparoscopic surgery (SILS + 1) which includes an additional port in the right-lower quadrant, has received growing interest in recent years. Our previous study [13] has demonstrated that SILS + 1 is safe and feasible for sigmoid colon and upper rectal cancer and has better short-term outcomes compared with CLS, including greater cosmetic benefits, less postoperative pain without compromising oncologic treatment principles. In cancer therapy research, the disease-free survival (DFS) and overall survival (OS) are the most important measurements in regard to long-term prognosis. Though there were a few retrospective studies and an RCT has reported the comparable long-term results of SILC for colon cancer, to date there no retrospective studies or RCTs investigating the long-term effects of SILS + 1 [14–17]. Thus, survival data from RCTs are needed to confirm the long-term oncological outcomes of SILS + 1 in the treatment of colorectal cancer.

We performed an RCT of CLS versus SILS + 1 in patients with rectosigmoid cancer and previously reported the short-term results. Thus, the aim of this study is to assess the long-term oncological efficacy after SILS + 1 or CLS for rectosigmoid cancer.

## Methods

### Trial design and patients

This study was an open-labeled, single-center, randomized, controlled, noninferiority trial, conducted in the Department of General Surgery, Nanfang Hospital, Southern Medical University, Guangzhou, China. This trial was registered in ClinicalTrials.gov on 21/04/2014 (NCT02117557). The Ethics Committee of Southern Medical University approved the trial (Reference number: NFEC-2014-026), and the protocol of this trial was published previously [18]. Written informed consent was obtained from all patients before they were enrolled in this study.

The inclusion criteria, exclusion criteria, and withdrawal criteria were included in our previous protocol. Patients were included if they were 18 to 80 years old; had histologically confirmed rectosigmoid cancer; diagnosed cT1-4aN0-2M0 lesions by abdominal CT and colonoscopy or EUS according to the 7th Edition of the AJCC Cancer Staging Manual; had tumors sized 5 cm or less and located in the rectosigmoid (defined as 10 to 30 cm from the anal verge, measured via colonoscopy or EUS). Patients who have complications requiring emergency operations, malignant disease within 5 years, or other conditions that affect the operation of abdominal surgery (BMI > 30 kg/m^2^, pregnant or previous abdominal surgery) were excluded.

### Endpoint

The primary endpoint of this trial was 3-year DFS. The DFS defined as the time from the date of randomization to the date of first confirmed recurrence or death from rectosigmoid cancer. The secondary endpoints were 5-year OS, early morbidity, operative outcomes, pathological outcomes, postoperative inflammation and immune response, postoperative recovery, pain intensity, and cosmetic results. The OS will be calculated from the date of randomization to the date of death from any cause.

### Surgical procedure

Both SILS + 1 and CLS were complied with the same principles of the operative extent by surgeons who had completed over 100 successful CLS cases and at least 10 successful SILS + 1 cases.

For the SILS + 1 procedure, a multiport device (SURGAID MEDICAL; XIAMEN, CHINA) was placed at a 5-cm periumbilical transverse incision and an additional 12-mm trocar was placed in the right lower quadrant served as the surgeon’s dominant operating channel. After mobilization, the specimen was retrieved through the primary incision where the SILS device was placed. After surgery, a drainage tube was inserted through the trocar incision to drain the pelvic cavity if the patients underwent anterior resection. The decision to add trocar(s) was made at the surgeon’s discretion and was defined as conversion to multiport surgery.

The CLS procedure was performed using 5 trocars placed in the regular position as described previously. After mobilization, the specimen was extracted through the umbilical incision which was transversely extended to 4-5 cm according to the tumor size. Once the length of the minilaparotomy exceeded 10 cm, laparoscopic surgery was considered as the conversion to open surgery.

For both approaches, surgical quality control was maintained by using mandatory intraoperative photographs that identified specific surgical fields, the resection margin of the specimen, and the abdominal incision. Five photos were used to verify the surgical quality, as follows: [1] high ligation in the root of inferior mesenteric artery and inferior mesenteric vein, [2] the macroscopic quality of the complete mesocolic excision, and [3] proximal and distal margin lengths over 5 cm. These photos were reviewed, and feedback was regularly provided to the investigators.

### Follow up

All participants were followed up regularly, and follow-up data, including recurrence and death, were recorded. Recurrence was identified by medical history and physical examination in combination with imaging evaluation and tissue biopsy by colonoscopy. Both groups of participants were followed up at 1 and 3 months after surgery, then every 3 months for the first 2 years and every 6 months for the next 3 years and then annually. Patients with tumor recurrence were followed up every 3 months until the date of the last follow-up examination.

### Randomization and data management

The sample size was determined by the early morbidity. According to the non-inferiority design, this analysis was based on an alpha of 0.025, a power of 80%, and a margin delta of 20%; a sample of at least 90 participants per group was calculated using the NCSS-PASS (11th edition, NCSS, LLC, Utah, USA). Assuming a 10% drop-out rate, the total number of participants needed per group was 99. Patients were randomized to undergo SILS + 1 or CLS according to a computer-generated randomization list at a 1:1 ratio. A research coordinator gave the surgeon the patients’ randomization numbers and group assignment in identical, opaque, sealed envelopes the day before surgery. All data were recorded in the case report form (CRF) by a research coordinator and reviewed by another coordinator. The CRF were double-checked to ensure the accuracy of the data before it was transferred into the trial database. An investigator reviewed the database to ensure accurate data collection using descriptive statistics to check for missing data and out-of range values. Any unclear data will be traced to the original medical records.

### Statistical analysis

Descriptive statistics were applied for baseline characteristics analyses. For categorical variables, including the primary outcome, a *χ*2 test or Fisher’s exact test was applied. For continuous variables, Student’s *t* test or the Mann–Whitney *U* test was applied. All analyses of disease-free survival and overall survival were performed using conventional 2-tailed superiority hypothesis tests with α = 0.05 and with 2-sided 95% CIs. The overall survival and disease-free survival were calculated using the Kaplan-Meier method. The log-rank test was used to do univariate comparisons. Multivariable mixed-effects cox regression was used to estimate the Hazard Ratios (HRs) and effects between the two groups. All statistical analyses were performed using SPSS 25.0 for Windows (SPSS, Inc., Chicago, IL, USA) and a two-sided P < 0.05 were considered statistically significant.

## Result

### Study population

**Fig. 1:**
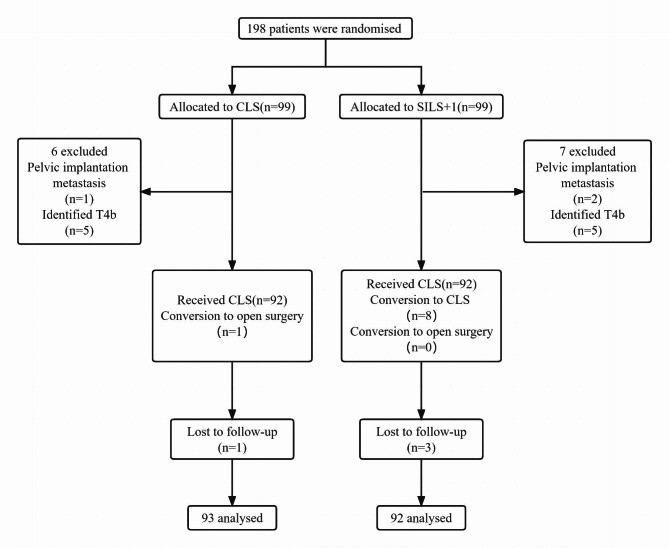
Consort diagram. SILS + 1, single-incision plus one-port laparoscopic surgery; CLS, conventional laparoscopic surgery

From April 2014 and July 2016, a total of 198 patients were randomly assigned to the SILS + 1 group or the CLS group (99 per group) (Fig. 1). 7 patients from SILS + 1 group (2 patients with pelvic implantation metastasis and 5 patients who had pathologic T4b tumors) and 6 patients from CLS group (1 patient with pelvic implantation metastasis and 5 patients who had pathologic T4b tumors) were excluded. Thus, the primary analysis set consisted of 185 patients (92 in the SILS + 1 group and 93 in the CLS group). The per-protocol population consisted of 176 patients, with 84 in the SILS + 1 group (92 patients in the primary analysis set minus 8 patients who did not adhere to their treatment plans) and 92 in the CLS group (93 patients in the primary analysis set minus 1 patient who did not adhere to his treatment plan). As our previous data showed that there are 8 patients with conversion to CLS in SILS + 1 group and 1 patient with conversion to open surgery in CLS group, the as-treated population consisted of 84 patients in the SILS + 1 group and 100 patients in the CLS group. The median follow-up period in the SILS + 1 group was 64.0 months (SD 14.7; range 7–79), and in CLS group was 65.0 months (SD 16.2 ; range 10–80 ), with a total of 4 patients (2.2%) lost to follow-up (3 in the SILS + 1 group and 1 in the CLS group, P = 0.37). The baseline of clinicopathologic characteristics of patients was shown in Table 1. The two groups were balanced regarding age, body mass indexes, comorbidities, and tumor location. The surgical procedure and outcomes are summarized in Table 2. The tumor diameter, number of lymph nodes harvested, pathological stage, and rate of adjuvant chemotherapy were similar between the 2groups.

**Table 1:** Patient baseline characteristics

Characteristics	CLS (n = 93)	SILS + 1(n = 92)	P
Age (years)	57.2 ± 11.7	56.9 ± 11.5	0.865
Gender			0.374
Male	56 (60.2)	49 (53.3)	
Female	37 (39.8)	43 (46.7)	
BMI (kg/m2)	23.0 ± 3.1	22.8 ± 2.7	0.722
ECOG status			0.139
0	72 (77.4)	79 (85.9)	
1	21 (22.6)	13 (14.1)	
ASA grade			0.94
I	53(57.0)	53 (57.6)	
II	35 (37.6)	34 (37.0)	
III	5 (5.4)	5 (5.4)	
Tumor location			
Sigmoid colon	17 (18.3)	28 (30.5)	0.061
Rectosigmoid	39 (41.9)	36 (39.1)	0.765
Superior rectum	37 (39.8)	28 (30.4)	0.218

Abbreviation: CLS, conventional laparoscopic surgery; SILS + 1, single-incision plus one-port laparoscopic surgery; BMI, body mass index; ECOG Eastern Cooperative Oncology Group; ASA, American Society of Anesthesiologists;

**Table 2:** Surgical Procedure and Pathologic Outcomes

Characteristics	CLS (n = 93)	SILS + 1(n = 92)	P
Surgical approaches			0.432
Sigmoidectomy	58 (62.4)	64 (69.6)	
Anterior resection	32 (34.4)	24 (26.1)	
Left hemicolectomy	3 (3.2)	4 (4.3)	
Additional trocar	0	8(8.7)	
Additional 1		1(1.1)	
Additional 2		2(2.2)	
Additional 3		5(5.4)	
Conversion to open surgery	0	1(1.1)	0.497
Harvested no. of LN	23.0 ± 11.2	20.9 ± 13.1	0.241
Pathologic TNM stage			0.148
0–I	18 (19.3)	20 (21.5)	
II	34 (36.6)	43 (46.7)	
III	41 (44.1)	29 (31.5)	
Pathologic T stage			0.939
Tis/T1	12 (12.9)	10 (10.9)	
T2	11 (11.8)	13 (14.1)	
T3	8 (8.6)	10 (10.9)	
T4a	62 (66.6)	59 (64.1)	
Pathologic N stage			0.241
N0	52	63	
N1	26	24	
N2	14	5	
Adjuvant chemotherapy	34(36.6)	28(30.4)	0.378

Continuous variables are described as the mean ± standard deviation (range); categorical variables are described as n (%)

Abbreviation: TNM, tumor-node-metastasis; LN, lymph node,

### The primary endpoint: disease-free survival

#### Primary analysis set

**Fig. 2:**
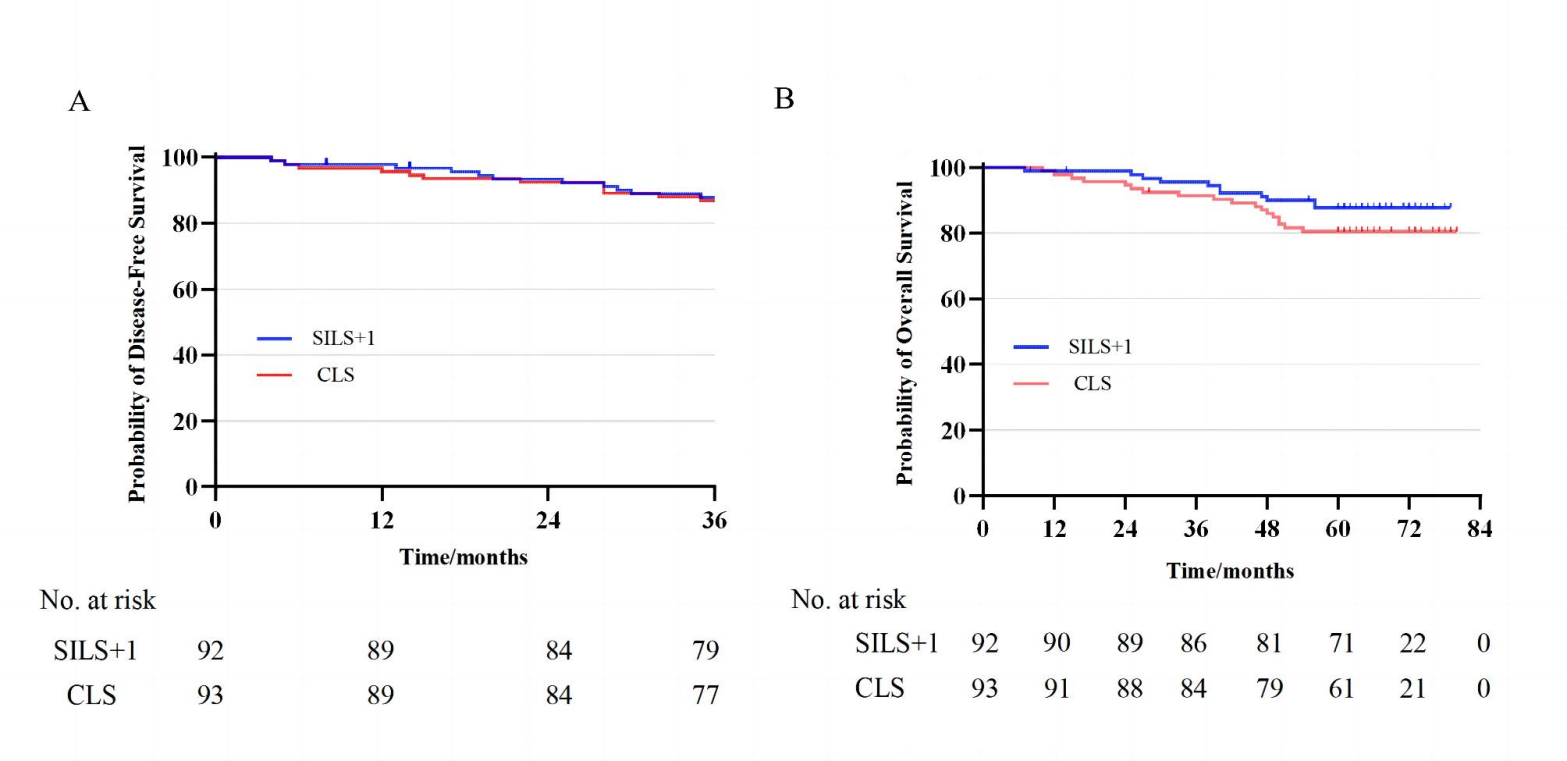
**A)** 3-year Disease-free survival; **B)** 5-year Overall survival. SILS + 1, single-incision plus one-port laparoscopic surgery; CLS, conventional laparoscopic surgery

The 3-year DFS rates were 87.8% (95% CI, 81.6–94.8%) in SILS+1 group and 86.9% (95% CI, 81.3–94.5%) in CLS group, with an absolute difference of 0.9% (95% CI, -8.7%to 5.8%) that did not exceed the prespecified noninferiority margin of -10% (Fig. 2a). The HR for 3-year DFS in the SILS + 1 group compared with that in the CLS group was 1.09 (95% CI, 0.48–2.47; P = 0.84) in the univariate Cox regression analysis. A similar HR was observed after adjusting for age, sex, T stage, and N stage (SILS + 1 vs. CLS HR, 0.91; 95% CI, 0.40–2.08; P = 0.82) (Table 3).

**Table 3:** Univariate and Multivariable Cox Regression Analyses of Risk Factors for Survival

	Univariate		Multivariable	
	3 Years Disease-free Survival Outcomes			
	HR (95%)	P	HR (95%)	P
Procedure (SILS + 1 vs. CLS)	1.36(0.62–2.96)	0.77	1.10 (0.49–2.41)	0.82
Sex(Male vs. Female)	1.25(0.57–2.76)	0.58	0.96(0.43–2.12)	0.91
Age(≤60 vs. >60)	0.89(0.41–1.94)	0.73	1.08(0.49–2.37)	0.84
T stage(T1 + T2 vs. T3 + T4)	4.32(1.02–18.28)	0.047	2.51(0.58–10.89)	0.22
N Stage (N0 vs. N+)	8.22(3.10-21.83)	<0.001	7.10(2.61–19.29)	<0.001
	5 Years Overall Survival Outcomes			
	HR (95%)	P	HR (95%)	P
Procedure (SILS + 1 vs. CLS)	1.53(0.74–3.18)	0.25	1.29(0.61–2.71)	0.50
Sex(Male vs. Female)	1.02(0.49–2.11)	0.94	0.79(0.38–1.64)	0.53
Age(≤60 vs. >60)	0.95(0.46–1.95)	0.88	1.11(0.54–2.31)	0.77
T stage(T1 + T2 vs. T3 + T4)	2.35(0.82–6.72)	0.11	1.39(0.47–4.11)	0.56
N Stage (N0 vs. N+)	6.61(2.84–15.42)	<0.001	6.24(2.59–15.04)	<0.001

Abbreviation: HR, hazard ratio

#### Per-protocol and as-treated populations

In the per-protocol analysis, the 3-year disease-free survival rates were 89.1% (9 of 84) patients who died or had a recurrence calculated by time to event in the SILS + 1 group and 86.8% (12 of 92) in the CLS group, with an absolute difference of 2.3% (1-sided 97.5% CI, − 7.3–7.2%). In the as-treated analysis, the 3-year disease-free survival rates were 89.1% (9 of 84) in the SILS + 1 group and 85.8% (14 of 100) in the CLS group, with an absolute difference of 3.3% (1-sided 97.5% CI, − 6.3–8.2%).

### Secondary outcomes

#### Overall survival

At the last follow up, 29 patients (15.7%) had died (11 in the SILS + 1 group and 18 in the CLS group) (Table 4). The 5 year OS rates were 86.7% (95% CI,79.6–93.8%) for SILS+1 group and 80.5% (95% CI,72.5–88.5%) for CLS group, with no statistical difference between the two groups (log-rank P = 0.25) (Fig. 2b). The difference in 5-year OS rate was 6.2% [95% CI, -4.4%to 11.6%] that did not cross the prespecified noninferiority margin of − 10%. The HR for all-cause mortality in the SLS + 1 group compared with that in the CLS group was 1.53 (95% CI, 0.74–3.18; P = 0.25) in the univariate Cox regression analysis. This estimate remained similar after controlling for age, sex, T stage, and N stage (SILS + 1vs CLS groups HR, 1.29; 95% CI, 0.61–2.71; P = 0.50) (Table 3).

**Table 4:** Recurrence Patterns

Variable	CLS (n = 93)	SILS + 1 (n = 92)	P
Total recurrence	20(21.5%)	14(15.2%)	0.27
Local Recurrences	2(2.2%)	1(1.1%)	0.19
Distant Recurrences			
Liver	3(3.2%)	6(6.5%)	0.48
Lung	9(9.7%)	6(6.5%)	0.43
Bone	2(2.2%)	2(2.2%)	1
Peritoneal dissemination	4(4.3%)	7(7.6%)	0.34
Total death	18(19.3%)	11(12.9%)	0.24
Rectosigmoid Cancer Related	15(16.1%)	10(10.9%)	0.29
Other	3(3.2%)	1(2.1%)	1

#### Recurrence

Within 5 years of follow-up, recurrence was recorded in 14 patients (15.2%) in the SILS + 1 group and 20 patients (21.5%) in the CLS group; the difference was not statistically significant (P = 0.27) (Table 4). Among 92 cases of SILS + 1 group, 6 (6.5%) had liver metastasis, 6 (6.5%) had lung metastasis, 7 (7.6%) had peritoneal dissemination, and 2 (2.2%) had bone metastasis, similarly, for 93 cases of CLS group, 3 (3.2%) had liver metastasis, 9 (9.7%) had lung metastasis, 4 (4.3%) had peritoneal dissemination, and 2 (2.2%) had bone metastasis. Regarding the local recurrence, these two groups were also similar. Cancer-related deaths were found in 10 (10.9%) patients in SILS + 1 group and 15 (16.1%) patients in CLS group, likewise, with no significant difference (P = 0.295) (Table 4).

### Subgroup analysis

**Fig. 3:**
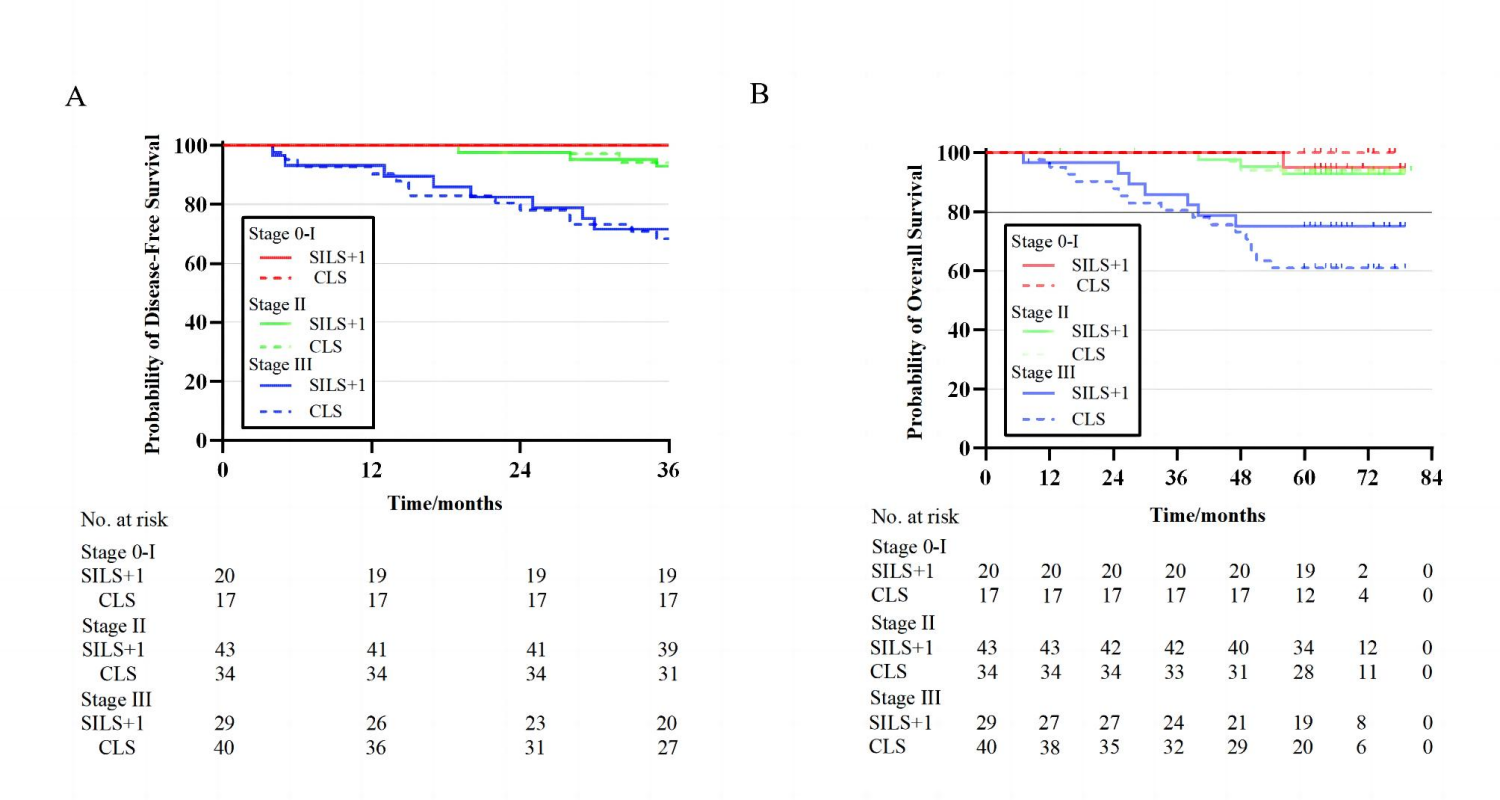
**A)** Kaplan-Meier Curves of 3-year disease-free survival by Pathologic Stage. **B)** Kaplan-Meier Curves of 5-year overall survival by Pathologic Stage. SILS + 1, single-incision plus one-port laparoscopic surgery; CLS, conventional laparoscopic surgery

The subgroup analysis revealed no significant differences in 3-year DFS rates between the SILS + 1 and CLS groups for any subgroup: for patients with pathologic stage 0-I were 100% vs. 100%; for stage II, 93.0% vs. 94.0% (log-rank P = 0.83); and for stage III, 71.6% vs. 74.4% (log-rank P = 0.83) (Fig. 3a). The 5-year overall survival rates for the SILS + 1 and CLS groups, among patients with pathologic stage I was 95.0% vs. 100.0% (log-rank P = 0.34); stage II, 90.5% vs. 93.9% (log-rank P = 0.60); stage III, 75.1% vs. 60.0% (log-rank P = 0.24) (Fig. 3b). Interaction tests showed that the differences in DFS and OS between the 2 groups did not significantly differ across the stages (all interaction, P > 0.05).

## Discussion

This randomized clinical trial conducted at Nanfang Hospital in China among patients with rectosigmoid cancer (clinical stage T1-4aN0-2M0), found that the 3-year disease-free survival and 5-year overall survival of patients assigned to the SILS + 1 group was not inferior to that of patients assigned to the CLS group. Additionally, no significant differences were found between the groups in the pattern of recurrence over the 5-year period.

Compared with CLS, SILC has been shown to achieve less postoperative pain, better cosmetic effect and lower risk for surgical site complications. Despite the encouraging results, SILC has not been widely adopted due to the technical challenges, including conflicts of surgical devices, loss of triangulation, in-line viewing and a significant learning curve distinct from traditional laparoscopy [19]. Thus, SILS + 1 with an additional port was attempted to overcome the above obstacles while maintaining the minimally invasive effect [20, 21]. In this present trial, we only enrolled patients with sigmoid colon and upper rectal cancer because the surgical approaches of these tumor sites were relatively simple, which did not require splenic flexure mobilization and mobilization of the rectum outside the peritoneum reflection. According our previous study, the learning curve of SILS + 1 is relatively short and comprised only 14 SILS + 1 procedures for experienced laparoscopic surgeons [22]. Similar to our previous retrospective study [19], Song et al. [23] and Yu et al. [24] have also demonstrated the surgical safety of SILS + 1 for colorectal cancer. In addition, our safety analysis data from the present study [13] showed that the short-term surgical outcomes including postoperative morbidity, mortality, and complication rates were similar between the CLS and SILS + 1 groups for patients with sigmoid colon and upper rectal cancer whereas SILS + 1 group could achieve shorter operation time, shorter surgical incision, and less postoperative pain.

Besides the favorable short-term outcomes, long-term oncological outcome is another important measurement for a new surgical technique in the field of radical cancer resection. Previously published observational studies and similar-scale randomized trials [16, 17] have reported that neither 3-year DFS nor 5-year OS were significantly different between SILS and CLS groups. However, to this date, there have been no reports concerning the long-term results of SILS + 1 for colorectal cancer. This is the first reportedly randomized controlled study, which confirmed the comparable long-term outcomes between the SILS + 1 and CLS for sigmoid colon cancer and upper rectal cancer. The current randomized clinical trial found that the 3-year disease-free survival of patients assigned to the SILS + 1 group was similar to that of patients assigned to the CLS group. In addition, the 5-year overall survival and recurrence patterns did not significantly differ between the two groups, either. Although the results were not statistically significant, patients in the CLS group tended to show a worse survival than the SILS + 1 group patients in this RCT, different from initial expectations. There are several plausible explanations as following. Many studies have previously reported that lymph node metastasis was a risk factor for local recurrence and poor prognostic in sigmoid colon or rectal cancer [25–28]. We speculate that the trend of higher lymph node metastasis rate in CLS group than the SILS + 1 group (43.0% vs. 31.5%), though without statistical significance, might contributed to a worse survival in the CLS group. Moreover, 3 patients died of diseases other than rectosigmoid cancer in the CLS group compared with only 1 patient in the SILS + 1 group, which might result in the lower 5-year OS rate in the CLS group than that of SILS + 1 group. Thus, we propose that differences in survival rates are not due to variations in the technical procedures but rather the differences in patient heterogeneity since both SILS + 1 and CLS in this trial were complied with the same principles of the operative extent.

Although the purpose of this study was to compare the effectiveness of SILS + 1 and CLS for colorectal cancer, we found 5-year OS rates and 3-years DFS rates of this present study were lower than those of other previously published studies. Watanabe et al [17] reported the survival data of a RCT study of multi-port laparoscopic colectomy (MPC) versus SILC in colon cancer surgery and showed no significant differences in 5-year recurrence-free survival (SILS vs. MPC: 88.0% vs. 91.0%, P = 0.479) and 5-years overall survival (SILC vs. MPC: 93.0% vs. 95.0%, P = 0.568) between the two group. In another propensity-score matched study [16] by Suzuki et al. also reported similar oncological outcomes between SILC group and MPC group with 5-year cancer-specific survival of 93.7% in SILC group and 93.3% in MPC group (P = 0.5278) and 3-year disease-free survival of 94% in SILC group and 93.2% in MPC group (P = 0.2829). However, the 3-year DFS of the present trial were 87.8% in SILS+1 group and 86.9% in CLS group, and the 5-year OS were 86.7% in SILS+1 group and 80.5% in CLS group. A reasonable interpretation of these findings is that the patients included in our RCT consisted of more pathologic stage III cases. Our subgroup analyses showed patients with pathologic stage III disease had worse DSF (SILS + 1 vs. CLS, 71.6% vs. 74.4%) and OS (SILS + 1 vs. CLS, 75.1% vs. 60.0%) compared to those of other pathologic stage patients. Similar to the present trial, Watanabe et al. also reported patients who were clinical stage III tended to show a worse survival in the SILS group [17]. In addition, in the JCOG0404 trial, which is an RCT of laparoscopic surgery versus open surgery for stage II or III colon cancer, patients with T4 or N2 disease also tended to have a poor prognosis in the laparoscopic group [29]. Thus, the long-term oncologic results of this study were comparable to these reports. We therefore believe, with good reasons, SILS + 1 could offer similar long-term outcomes compared with CLS. Taken together, the short-term and long-term results of this study suggest that in the setting of sigmoid colon and upper rectal cancer, SILS + 1 is noninferior to CLS when performed by expert surgeons at high volume referral centers in China.

This study has several limitations. First, we only enrolled patients with sigmoid colon cancer or upper rectal cancer and therefore applying SILS + 1 for patients with other different site of colon cancer needs to be verified through other clinical trials. Second, although the possibility of allocation bias was reduced using random principle, loss of follow-up after operation might have affected this study. Third, this trial was a single-institutional RCT, the number of cases was limited because the sample size was calculated based on the early morbidity rate. Thus, a further prospective multi-institution RCT with larger number of patients will be required to confirm the long-term survival of SILS + 1 and whether or not SILS + 1 is indeed a viable alternative strategy to CLS for colorectal cancer.

## Conclusion

In conclusion, we found no significant difference in 3-year DFS and 5-year OS of patients with sigmoid colon and upper rectal cancer treated with SILS + 1 vs. CLS by experienced surgeons. Together with our previous reported short-term outcomes, these long-term oncologic outcomes of SILS + 1 support the adoption of this procedure as an alternative treatment for CLS in rectosigmoid cancer. It might be more practical to apply SILS + 1 over pure SILS.

## Data Availability

The datasets used and analysed during the current study are available from the corresponding author on reasonable request.

## Abbreviations

CLS: Conventional laparoscopic surgery
RCT: Randomized clinical trial
SILC: Single-incision laparoscopic colectomy
SILS + 1: Single-incision plus one-port laparoscopic surgery
DFS: Disease-free survival
OS: Overall survival
Case: Report form CRF
HR: Hazard Ratio
